# Blunted diurnal interleukin-6 rhythm is associated with amygdala emotional hyporeactivity and depression: a modulating role of gene-stressor interactions

**DOI:** 10.3389/fpsyt.2023.1196235

**Published:** 2023-05-30

**Authors:** Yuko Hakamata, Hiroaki Hori, Shinya Mizukami, Shuhei Izawa, Fuyuko Yoshida, Yoshiya Moriguchi, Takashi Hanakawa, Yusuke Inoue, Hirokuni Tagaya

**Affiliations:** ^1^Department of Clinical and Cognitive Neuroscience, Toyama University School of Medicine, Toyama, Japan; ^2^Department of Health Science, Kitasato University School of Allied Health Sciences, Sagamihara, Kanagawa, Japan; ^3^Department of Behavioral Medicine, National Institute of Mental Health, National Center of Neurology and Psychiatry, Tokyo, Japan; ^4^Department of Radiological Technology, Kitasato University School of Allied Health Sciences, Sagamihara, Kanagawa, Japan; ^5^Occupational Stress and Health Management Research Group, National Institute of Occupational Safety and Health, Kawasaki, Kanagawa, Japan; ^6^Integrative Brain Imaging Center, National Center of Neurology and Psychiatry, Tokyo, Japan; ^7^Department of Integrated Neuroanatomy and Neuroimaging, Kyoto University, Kyoto, Japan; ^8^Department of Diagnostic Radiology, Kitasato University School of Medicine, Sagamihara, Kanagawa, Japan

**Keywords:** interleukin-6, depression, basolateral nuclear complex, magnetic resonance imaging, dorsolateral prefrontal cortex, single nucleotide polymorphism, circadian rhythm, immune system

## Abstract

**Background:**

The immune system has major roles in the brain and related psychopathology. Disrupted interleukin-6 secretion and aberrant amygdala emotional reactivity are well-documented in stress-related mental disorders. The amygdala regulates psychosocial stress-related interleukin-6 affected by related genes. These led us to comprehensively examine the relationship between interleukin-6, amygdala activity, and stress-related mental symptoms under gene-stressor interactions.

**Methods:**

One hundred eight nonclinical participants with various levels of anxiety/depression underwent magnetic resonance imaging scans during an emotional face task for amygdala activity and saliva collection (at 10-time points across 2 days) for the total output and diurnal patterns of interleukin-6. Gene-stressor interactions between rs1800796 (C/G) and rs2228145 (C/A) and stressful life events for the biobehavioral measures were explored.

**Results:**

The blunting of interleukin-6 diurnal pattern was associated with hypoactivation of the basolateral amygdala in response to fearful (vs. neutral) faces (*t* = 3.67, FWE-corrected *p* = 0.003), and was predominantly observed in individuals with rs1800796 C-allele homozygotes and negative life changes in the past year (*F* = 19.71, *p* < 0.001). When considered in a comprehensive model, the diminished diurnal pattern predicted greater depressive symptoms (*β* = −0.40), modulated by the amygdala hypoactivity (*β* = 0.36) and rs1800796-stressor interactions (*β* = −0.41; all *p* < 0.001).

**Conclusion:**

Here we show that the blunted interleukin-6 diurnal rhythm predicts depressive symptoms, modulated by amygdala emotional hyporeactivity and gene-stressor interactions. These findings indicate a potential mechanism underlying vulnerability to depressive disorders, suggesting their early detection, prevention, and treatment through the understanding of immune system dysregulation.

## 1. Introduction

The immune system has major roles in the brain and related mental health conditions. Despite the mechanisms of their link remaining unknown, previous research has indicated that extreme stress can disrupt the immune system regulation (1). For example, excessive secretion of glucocorticoids causes dendritic shrinking and eventually neuronal death in stress-related brain regions such as the hippocampus and prefrontal cortex (PFC) (2) via its inducing glutamate release (3) and thereby N-methyl-D-aspartate (NMDA) receptor overstimulation (4, 5), while it elicits dendritic lengthening in the amygdala (particularly its basolateral part) (6). In addition, the exaggerated glucocorticoid secretion produces oxidative stress in the long term via free radical formation through mitochondrial dysfunction (1). These detrimental effects on the organism can activate the immune system (7). Hence, the immune system can be disrupted when exposed to excessive stressors, possibly leading to mental disorders (8–10).

Although peripheral inflammatory cytokines do not readily pass through the blood–brain barrier (BBB), they can penetrate the central nervous system under specific conditions, such as BBB alteration (11, 12), causing neuroinflammation through glial cell activation (13). Glial cells can become activated by stress as well as infection (14), specifically in the amygdala when exposed to psychosocial stressors (15, 16). The amygdala modulates behavioral and physiological responses to stressors (1), determining their emotional significance (17). Furthermore, the amygdala regulates the expression of stress-related inflammatory cytokines (18, 19) as well as corticotropin-releasing factors (20), suggesting its critical role in the immune system and stress-related mental disorders.

Supporting this idea, immune system perturbation and aberrant amygdala emotional reactivity have been reported in stress-related mental disorders. Previous functional magnetic resonance imaging (fMRI) studies have shown that in patients with major depressive disorder (MDD) (21–23) and posttraumatic stress disorder (PTSD) (24–27) as compared to healthy controls, the amygdala primarily exhibits stronger responses to emotionally negative (vs. neutral) stimuli, whereas its dorsal and lateral parts exhibit significantly weaker responses simultaneously (23, 25). Dysfunctional activity during emotional processing has been also found in the hippocampus and PFC in PTSD (25, 26, 28) and MDD (21–23) with some inconsistent findings (29), whose regions are implicated in cognitive deficits such as in learning, memory, and decision making (30, 31). Moreover, previous immunological studies have demonstrated increased levels of IL-6 and C-reactive protein (CRP) but not IL-1β or tumor necrosis factor (TNF)-α (32) in MDD, and increased levels of these markers in PTSD (33, 34). Above all, disrupted IL-6 secretion has been commonly observed and thus conceivably underlies the pathophysiology of stress-related mental disorders.

IL-6, which is primarily secreted by T cells, macrophages, and adipocytes, can function as either an anti-or pro-inflammatory cytokine (35), playing a primary role in inflammation (36). IL-6 expression is regulated by related genes and their interactions with stressors (37, 38). In particular, representative single nucleotide polymorphisms (SNPs), including rs1800795/6 (*IL6*: C/G) and rs2228145 (IL-6 receptor, *IL6R*: C/A) (39, 40), impact circulating IL-6 levels (41, 42), such that the C substitution of *IL6* is reported to be associated with lower IL-6 levels (40, 43) while that of *IL6R* is associated with higher IL-6 levels (42, 44). Moreover, IL-6 levels possess a clear diurnal rhythm characterized by two peaks and troughs during a 24-h period (45, 46). In particular, the pattern of an evening trough to a midnight peak is predominantly observed in both saliva and blood (45–48). Plasma and saliva IL-6 levels are weakly-to-moderately correlated (49–51), despite the detection rate in the saliva being reportedly higher and more stable than that in the blood (52). We previously reported that individuals with a history of childhood abuse, a significant risk factor for developing stress-related mental disorders (53), exhibit a diminished salivary IL-6 diurnal rhythm (54). Similarly, flattened plasma IL-6 diurnal patterns have also been reported in individuals with combat-zone experience, regardless of the presence/absence of PTSD (55), suggesting that exposure to intense stressors potentially leads to disruption of the IL-6 diurnal patterns through allostatic overload (1). Thus, alterations in the diurnal patterns and total output of IL-6 may be the key factors underlying one’s vulnerability to stress-related mental disorders, through gene-stressor interactions.

Despite the lack of comprehensive research on the relationship between IL-6 levels and amygdala emotional reactivity in the context of depression/anxiety, several seminal studies have investigated the relationship between IL-6 levels and amygdala emotional reactivity in general. No significant difference was found in amygdala activity in response to emotional stimuli between a control group and a group with increased IL-6 levels, induced by the administration of a low-dose bacteria/toxin (56, 57). Similarly, no significant differences were observed in the relationship between the circulating IL-6 levels and amygdala emotional reactivity in healthy participants (58) and breast cancer survivors who completed treatment (59). By contrast, other studies focusing on psychosocial stress in individuals without apparent psychopathology demonstrated significant correlations between increased IL-6 levels and amygdala activity, as well as its functional connectivity (FC) with the dorsomedial PFC while receiving negative feedback (60). A significant interaction was also reported between a poverty stressor and amygdala emotional reactivity on a composite of inflammatory markers, including IL-6 (61). Accordingly, psychosocial stressors are likely to connect IL-6 secretion, amygdala emotional reactivity, and depression/anxiety through interacting with genetic factors.

Therefore, this study aimed to investigate the relationship between IL-6 levels, amygdala emotional reactivity, and depression/anxiety, considering gene-psychosocial stressor interactions, in a sizeable community sample with various levels of depression/anxiety. Specifically, we hypothesize that: (1) disrupted IL-6 secretion (i.e., increased total output or diminished diurnal rhythm) is associated with perturbed (i.e., increased or decreased) amygdala activity in response to emotional stimuli; (2) disrupted IL-6 secretion is correlated with greater depressive/anxiety symptoms; and (3) disrupted IL-6 secretion can be predicted by the interactions between psychosocial stressors and *IL6* or *IL6R* SNPs. Additionally, given the amygdala’s regulatory role in IL-6 secretion, we examine the pathways from the amygdala to IL-6 secretion and depression under gene-stressor interactions. Further, we explore correlations between IL-6 levels and FC throughout the brain.

## 2. Materials and methods

### 2.1. Participants

We recruited participants who did not receive a psychiatric diagnosis and psychotropic medication, since either considerably affects the levels of immune/inflammatory markers and amygdala emotional reactivity (62, 63). The individuals’ risk of developing stress-related mental disorders was assessed by measuring the current stress-related symptoms (i.e., depression/anxiety) to examine their relationships with the variables of interest based on the perspective of the dimensional model for mental disorders (64). This model postulates that the manifestation of mental disorders can be dimensional, but not categorical, including individuals with various levels of correspondent mental symptoms from none to moderate (i.e., risk individuals) and severe (i.e., patients) (64).

Participants were recruited via websites, magazine advertisements, and billboards at Kitasato University from people in the community who dwelled in Tokyo and surrounding cities such as Kanagawa, Chiba, Saitama, and Ibaraki prefectures. The eligibility criteria were as follows: (1) 18–59 years old; (2) no current Axis-I psychiatric disorders or substance-abuse history as determined by the 4th edition of the Diagnostic and Statistical Manual of Mental Disorders by an experienced clinician (YH); (3) no major medical illnesses; (4) no regular intake of psychotropics, steroids, or opioids; (5) no metal nor medical appliance on/inside the body; (6) no history of brain injury or trauma with loss of consciousness over 10 min; and (7) no habitual intake of medicine for pain or fever (e.g., loxoprofen or acetaminophen) or excessive caffeine (> 400 mg/day) that could affect the blood-oxygen-level-dependent (BOLD) signal (65). Of the 134 recruited participants, 11 did not meet the inclusion criteria due to a history of brain trauma with loss of consciousness (> 10 min, *n* = 1), epilepsy (*n* = 1), orthodontic appliances (*n* = 2), immune-related diseases (*n* = 3, including 1 hyperlipidemia, 1 systemic lupus erythematosus, and 1 ulcerative colitis case), and regular intake of loxoprofen for migraines (*n* = 4). In total, 123 participants provided written informed consent.

Additionally, this study shared the sample of our previous studies as follows: 50.0% (66), 75.9% (67), 76.8% (68), and 91.7% (54), although there was no overlap with Izawa et al. (46) and Hakamata et al. (69). Studies by Hakamata et al. (66–68) aimed to examine the relationship between resting-state FC and memory biases, and the study by Hori et al. (54) examined the relationship between childhood abuse and IL-6 levels. However, the current study focused on the relationship between IL-6 levels, depression, and amygdala activity during an emotional task in terms of gene-stressor interactions. Thus, the focus of the current study was entirely different from those of the previous studies.

This study was approved by the Kitasato University Medical Ethics Organization (C17-126 and G18-02 for genome) and the National Center of Neurology and Psychiatry Ethics Committee (A2018-064) as part of a research project to examine cognitive bias and related neurobiological mechanisms; and was conducted in compliance with ethical guidelines issued by the National Ministry of Health, Labor, and Welfare and Declaration of Helsinki.

### 2.2. Study procedures

Participants underwent psychological assessment and MRI scans on the same day; saliva was collected five times daily on two consecutive weekdays within 2 weeks of the initial assessment day. Saliva was also collected from a subset of the participants (*n* = 73) for DNA extraction. The sample attrition was because the genetic part of this study (G18-02) was approved by the ethics committee in June 2018, which was about 8 months after the main part started (C17-126, approved in October 2017). Participants provided the following information on potential IL-6 confounders: age, sex, body mass index (BMI), daily caffeine intake, smoking habits, monthly alcohol consumption, and years of education. For women, menstrual status information was also obtained. Scoring details of BMI, daily caffeine intake, monthly alcohol consumption, and menstrual status are described in Supplementary material S1.1.

### 2.3. Psychological assessment

#### 2.3.1. Depressive/anxious symptoms

Depressive symptoms over the past 2 weeks were assessed using the Beck Depression Inventory-II (BDI-II) (70, 71), comprising 21 items rated on a 4-point scale (from 0: “None” to 3, depending on severity). Scores ≥14 indicate the presence of significant depressive symptoms (70, 71). Additionally, subscales from the 54-item Hopkins Symptom Checklist (HSCL) (72, 73) were employed to assess anxiety (8 items) and depressive (13 items) symptoms in the past week; each item was rated on a 4-point scale (1: “None” to 4, depending on frequency).

#### 2.3.2. Psychosocial stressors

Psychosocial stressors were identified by assessing stressful life events experienced in the past year (e.g., divorce or separation from a partner, death of a family member, and taking a new job), using the Life Experiences Survey (LES) that comprises 57 items rated on a 7-point scale (from−3: “Extremely negative” to +3: “Extremely positive”) (74). We used the Japanese version of LES that has been standardized (75).

Scores were summed for negatively-and positively-rated life events, respectively, as negative and positive impact scores. These scores were treated as absolute values (74, 75). To assess the overall impact of the negative and positive life events that occurred in each individual, we used the balanced impact score calculated by subtracting the positive impact score from the negative impact score (74, 75). Values more than zero on this index indicate the negative life changes (NLC) experienced on the whole, while zero indicates no such NLC experienced.

According to Iwamitsu et al. (75), the balanced impact score has higher test–retest reliability (*r* = 0.65) than the negative impact score (*r* = 0.50) and positive impact score (*r* = 0.46; *p* < 0.01 for all). This score also has higher criterion-related validity: *r* = 0.40 with state anxiety assessed by Spielberger Trait Anxiety Inventory (STAI) (76) and *r* = 0.41 with depression by Self-Rating Depression Scale (SDS) (77) (*p* < 0.01 for both), as compared to negative impact score (*r* = 0.29 for STAI and *r* = 0.31 for SDS) and positive impact score (*r* = 0.10 for STAI and *r* = 0.08 for SDS) alone.

In the case that the balanced impact score did not follow a normal distribution, participants were classified into two groups for gene-stressor interaction analysis: those with NLC and without NLC. The classification was used for clarity, but results with this score as a continuous variable are also reported in Supplementary material S2.4 for reference.

### 2.4. Measurement of IL-6 levels

IL-6 levels were measured based on saliva collected five times daily over two consecutive typical weekdays: upon awakening (T1), 30 min after awakening (T2), midday (11:30–12:30) (T3), in the evening (17:30-18:30) (T4), and at bedtime (T5).

#### 2.4.1. Saliva collection procedures

Saliva collection was conducted as per the guidelines for best practices (78). An experimenter carefully explained and demonstrated saliva collection procedures for each participant, and each received written instructions describing the saliva collection procedures. The participant was encouraged to practice taking a saliva sample with the experimenter present. Specifically, participants were instructed to collect their saliva in a microtube by passively drooling for approximately 3 min and to concurrently access a customized web-entry form via their smartphone so that the exact time was automatically recorded whenever they collected saliva.

In addition, on the customized web-entry form, participants recorded the exact time they went to bed and woke up (i.e., sleep duration). They also rated their sleep quality ranging from 1 (did not sleep well) to 3 (slept well) upon awakening, their perceived stress ranging from 1 (did not perceive stress) to 4 (high-stress levels), and physical health conditions of the day ranging from 1 (bad) to 3 (typical) at bedtime. Further, women answered an extra question on their menstrual status at the starting day of saliva collection to validate their menstrual cycle provided on the assessment day.

Saliva was collected on two consecutive, typical weekdays using personalized kits with 20 color-coded tubes for each sample time (e.g., *blue* for bedtime), which were labeled with the date and time of measurement. Participants chose a typical weekday within 2 weeks after the initial assessment to begin collecting saliva. Also, for naturalistic measurement (i.e., under participants’ usual routine), the collection time of T1 and T5 were not specifically designated as long as the participant did not engage in shift work. They were instructed never to collect saliva on days when they would be engaged in any special activity or relaxing at home. During the two consecutive days, as well as the night before, the participants were required not to consume alcohol. Similarly, taking any food or drink besides water, exercising, tooth-brushing, and showering or bathing was not allowed 1 h before saliva collection.

In the cases required to collect saliva away from home (e.g., office or school), two freezer packs and a cooler bag were provided to each participant designed to keep the saliva refrigerated below 3°C for at least 8 h at an outdoor temperature of 25°C. Participants were instructed to store collected samples in their home freezers below −18°C as soon as possible when they returned home. Within 3 days of completing saliva collection, the samples were transported to the National Institute of Occupational Safety and Health in a frozen state (to author SI).

#### 2.4.2. IL-6 assay

For the salivary assay, slowly thawed samples were centrifuged (3,000 rpm, 1710-*g* force) for 10 min, and the concentration of IL-6 (pg/ml) was quantified by a Human IL-6 Quantikine HS ELISA (enzyme-linked immunoassay) Kit, 3rd Generation (R&D Systems, United States). Then, IL-6 levels at each time point were adjusted for total salivary protein levels at the corresponding time point (mg/ml) measured by a Bradford Protein Assay Kit (Bio-Rad Laboratories, Inc., United States) [i.e., IL-6 (pg/ml) divided by total protein (mg/ml)]. Intra-and inter-assay coefficients of variation for IL-6 were 4.4 and 9.9%, respectively. Detailed assay procedures are reported in our previous study (54).

#### 2.4.3. Calculation of IL-6 indices

##### 2.4.3.1. Total output

We calculated the area under the curve with respect to ground level (AUC_g_) based on the following standard formula (79): [(T1 + T2) × Time_T2-T1 (h)_/2] + [(T2 + T3) × Time_T3-T2 (h)_/2] + [(T3 + T4) × Time_T4-T3 (h)_/2] + [(T4 + T5) × Time_T5-T4 (h)_/2].

##### 2.4.3.2. Diurnal patterns

Izawa et al. (46) measured IL-6 levels at seven-time points and revealed that salivary IL-6 has a biphasic diurnal pattern, as seen with plasma IL-6, despite slight differences in observed time of peaks and troughs. In plasma, a peak tended to appear between 16:00 and 18:00, with a trough around 21:00; a second larger peak emerged from midnight to early morning, once again reaching a trough around 8:00 (45, 47, 48, 80). In contrast, saliva had a small peak at 16:00, a trough around 18:00–19:00, and another far larger peak appeared at midnight (46). Unlike in plasma, the high IL-6 levels at midnight were sustained in saliva upon waking (7:00–8:00). This is potentially due to the accumulation of inflammatory proteins secreted during the night in saliva since salivary protein levels are reportedly high at awakening (81). Thus, the IL-6 diurnal pattern was indexed by subtracting T4 from T5 levels to assess the increase from an evening trough to a midnight peak. Due to the high salivary IL-6 levels in the morning and the insufficient number of time points, Cosine or Fourier analysis was not applied to the current data. However, the results for other potential indices (e.g., T1–T4 and T5–T3) are also reported in Supplementary Table S3. The index values were averaged across both collection days to capture an individual’s basal IL-6 secretion patterns. Positive values indicate the presence of a diurnal increase, while negative and zero values indicate its absence (i.e., a diminished/blunted or disrupted pattern).

### 2.5. Magnetic resonance imaging scans

Anatomical and functional MRIs were acquired using a 32-channel phased-array head coil, 3.0 T scanner (Discovery MR750; GE Healthcare, United States). Structural images were acquired using a 3D T1-weighted sequence [slice thickness without slice gap = 1.0 mm, field of view (FOV) = 256 mm, matrix = 256 × 256, repetition time (TR) = 6.4 ms, echo time (TE) = 2.6 ms, inversion time (TI) = 400 ms, and flip angle (FA) = 14°]. For functional images, data were acquired using fast-gradient echo-planar T2*-weighted imaging with five dummy volumes at the beginning of the session. Each functional volume comprised 38 transverse slices (slice thickness = 3.0 mm, slice gap = 1.0 mm, FOV = 192 mm, matrix = 64 × 64, TR = 3,000 ms, TE = 30 ms, and FA = 90°).

For preprocessing and analyses of brain activity and FC, we used SPM12^1^ and CONN Functional Connectivity Toolbox, version 18b,^2^ respectively; both were operated on the Matlab 2019b platform.^3^

### 2.6. Functional magnetic resonance imaging preprocessing

For brain activity analysis, the following preprocessing procedures were performed using SPM12: realignment, slice timing correction, co-registration of the structural image to the functional images, segmentation of the T1 structural volume image, spatial normalization to the Montreal Neurological Institute (MNI) space, and Gaussian spatial smoothing [full width at half maximum (FWHM): 6 mm] (82). No excessive head motion (i.e., > 3 mm translation/rotation in any direction) was observed in the realignment.

For FC analysis, the aforementioned preprocessing procedures were similarly performed using a default pipeline with outlier detection (“scrubbing” to eliminate excessive head motion). In the outlier detection, three confounders were removed via principal-component-based noise-correction (“CompCor” method) (83): signal noise from the white matter and cerebrospinal fluid; within-subject covariates, including head-motion artifacts and scrubbing parameters; and the main condition effect convolved with a hemodynamic response function. The band-pass filter was set with a frequency window of 0.008–0.12 Hz. FWHM was 6 mm.

### 2.7. Functional magnetic resonance imaging experiment

**Figure 1:**
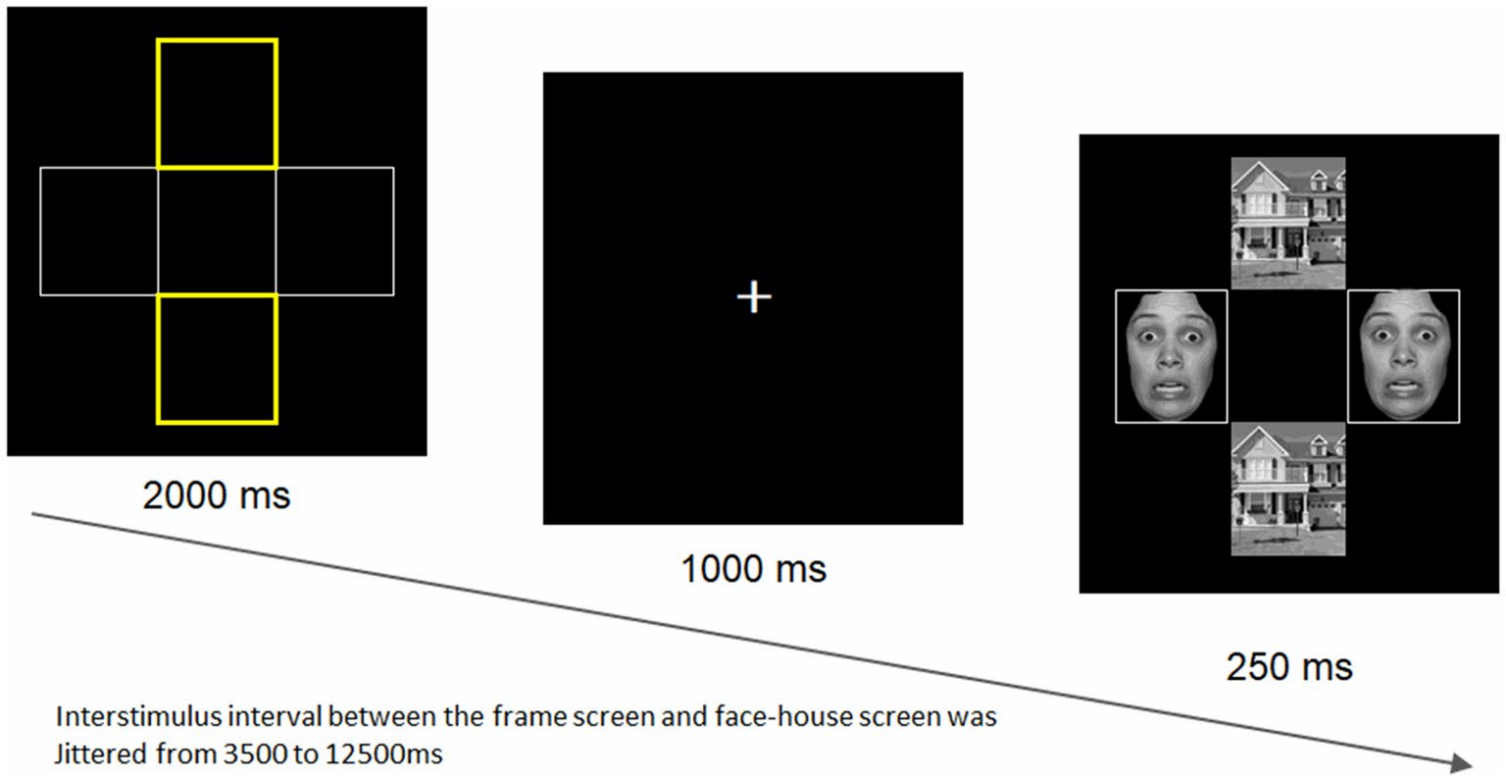
The face-house matching task. The task was created by Vuilleumier et al. (84). A total of 8 different facial stimuli (4 women and 4 men) were selected from the ATR database (DB99; ATR-Promotions, Inc., Kyoto, Japan). An example of a fearful facial stimulus borrowed from the NimStim set of facial expressions (86) is shown here, as the ATR prohibits the publication of facial stimuli. The stimulus intervals varied randomly between 3.5 and 12.5 s for a total of 48 trials, with a mean of 7.5 s. The task duration was 8 min 51 s, including the five dummy scans. The positions and emotional valences of faces were randomly presented. Both faces in each pair had the same facial expression on a given trial.

We employed a modified version of the face-house matching task (84), which is widely used to capture the amygdala activity in response to emotional stimuli (85), as previously reported (69). As illustrated in Figure 1, four frames are displayed for 2,000 ms, with either the two horizontal or vertical frames being highlighted in yellow. Then, after a fixation at the center of the screen presented for 1,000 ms, they were replaced by two faces (either neutral or fearful ones of the same individuals) in the location of the non-highlighted frames and two houses in the location of highlighted frames for 250 ms. Participants were instructed to always attend to the two highlighted frames and judge whether the subsequent houses are identical while faces are simultaneously appearing.

For face stimuli, we used eight different individuals’ faces with fearful and neutral expressions (four women and four men), selected from an established standard facial database for the Japanese population (DB99; ATR-Promotions, Inc., Kyoto, Japan). We used eight houses, as used in the original study (84). All pictures were black and white photographs (visual angle 18° × 13°) presented on a black background and projected through a mirror mounted onto the head coil. The stimulus presentation schedule was controlled by the E-prime 2.0 Professional software (Psychology Software Tools, Inc., Pittsburgh, PA) in synchronization with MR signals. The stimulus intervals varied randomly between 3.5 and 12.5 s for a total of 48 trials (mean: 7.5 s). The task duration was 8 min 51 s, including the five dummy scans. The positions and emotional valences of faces were randomly presented. To assess amygdala emotional reactivity, we compared responses to fearful vs. neutral faces.

### 2.8. DNA extraction and SNP genotyping

For genotyping, saliva samples were collected from each participant with an Oragene DNA Kit (DNA Genotek Inc., Canada). We specifically studied the rs1800796 (*IL6*) and rs2228145 (*IL6R*) SNPs. The *IL6* gene is located on chromosome 7p21, with the well-documented SNP, rs1800795 (−174G/C), located at its promoter region. However, the minor allele frequency of rs1800795 is known to be 0% in the Japanese population according to SNPedia (87). Therefore, we focused on rs1800796 (−572C/G) instead, which is also located in the promoter region of *IL6* in strong linkage disequilibrium with rs1800795. Like rs1800795, rs1800796 is known to also affect circulating IL-6 levels (23). The *IL6R* gene is located on chromosome 1q21, and its missense variant, rs2228145 (Asp358Ala; A/C), located at exon 9, maps to the IL-6R cleavage site.

Genomic DNA was purified from centrifuged saliva using the ethanol precipitation protocol and the prepIT.L2P manual purification protocol (DNA Genotek Inc., Canada). *IL6* rs1800796 (assay ID: C___11326893_10) and *IL6R* rs2228145 (assay ID: C___16170664_10) were genotyped using the TaqMan SNP Genotyping Assays. The polymerase chain reaction was carried out using GeneAce Probe qPCR Mixα (Nippon Gene, Japan) under the following conditions: 1 cycle at 95°C for 10 min followed by 45 cycles at 95°C for 15 s and 60°C for 1 min. The allele-specific fluorescence was measured with an ABI PRISM 7900 Sequence Detection System (Applied Biosystems, United States).

The numbers of participants with rs1800796 C/C, C/G, and G/G genotypes were 41 (56.2%), 24 (32.9%), and 8 (11.0%), respectively; and those with rs2228145 A/A, A/C, and C/C genotypes were 26 (35.6%), 37 (50.7%), and 10 (13.7%), respectively. The genotype frequency for these two SNPs did not deviate from Hardy–Weinberg equilibrium: *χ^2^*(1) = 2.20, *p* = 0.14, and *χ^2^*(1) = 0.31, *p* = 0.58, respectively. Due to the low frequency of minor alleles, participants were assigned to the following groups: “C/C” or “C/G + G/G” for rs1800796 and “C/C” or “A/A + A/C” for rs2228145.

### 2.9. Data analyses

#### 2.9.1. Relationships between IL-6 levels, amygdala emotional reactivity, and depressive/anxiety symptoms

**Figure 2:**
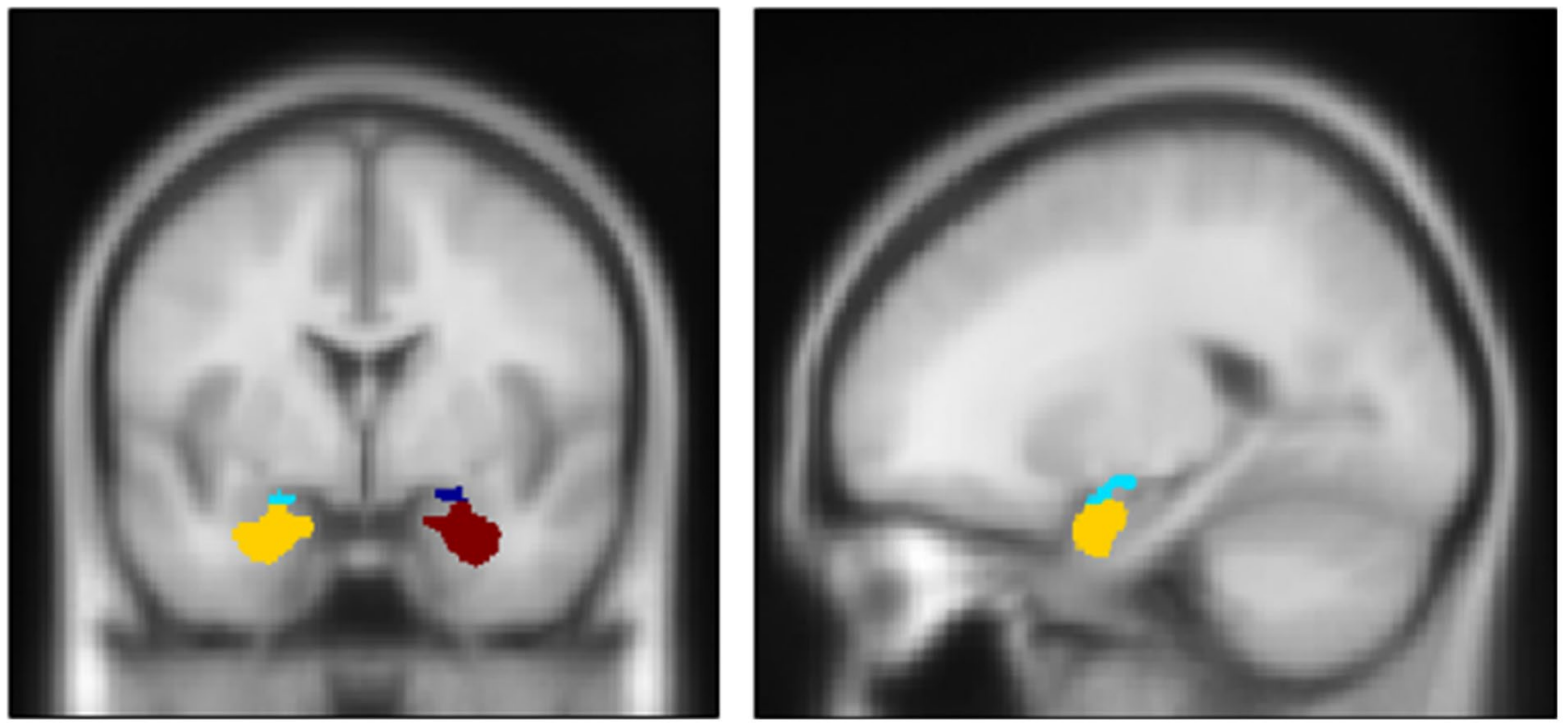
Anatomical definition of two major subnuclei of the amygdala complex: centro-median nucleus (CEM) and latero-basal nucleus (BLA). Dark blue: Right CEM. Red: Right BLA. Light Blue: Left CEM. Yellow: Left BLA. Center MNI coordinate and volume: (Right CEM) (23, −5, −14); 222 mm^3^; (Right BLA) (26, −1, −24), 1,699 mm^3^; (Left CEM) (−21, −6, −14), 345 mm^3^; (Left BLA) (−23, −3, −25), 1938 mm^3^. The definitions are based on Jüelich histological atlas (cyto-and myelo-architectonic segmentations: https://fsl.fmrib.ox.ac.uk/fsl/fslwiki/Atlases/Juelich).

After preprocessing fMRI data, we performed a 1st-level analysis using a general linear model to create a linear contrast map between the two conditions (i.e., fearful > neutral) for each participant. Then, using these contrast maps, we performed a 2nd-level analysis to explore regions exhibiting significant differences concerning measured IL-6 levels throughout the brain. For the amygdala, small volume correction (SVC) was applied to its main subnuclei, the basolateral nucleus (BLA) and centromedial nucleus (CEM), using the Jüelich histological atlas (Figure 2).^4^ The statistical threshold was set at *p* < 0.0125 (at peak level) for the amygdala (based on the number of bilateral subnuclei). For other brain regions, family-wise error (FWE)-corrected *p* < 0.05 (at peak level) was applied.

We further performed a correlation analysis to examine the relationship between amygdala activity, IL-6 indices, and depressive/anxiety symptoms, using the *β* values of the amygdala subnuclei that were extracted based on the anatomical mask of Jüelich atlas with MarsBaR version 0.43 (i.e., as the whole subnuclei values).^5^ Additionally, despite our primary interest in the amygdala, results of the hippocampus were also reported due to its adjacency to the amygdala. Statistical threshold was set at *p* < 0.0045 with the Bonferroni correction.

#### 2.9.2. Effects of gene-stressor interactions on IL-6 secretion

Next, a three-way analysis of covariance (ANCOVA) was conducted for the observed IL-6 index with SNPs (rs1800796 and rs2228145) and the NLC grouping as between-subject factors to determine whether gene-stressor interactions predict a disrupted IL-6 secretion pattern. If significant interaction effects were found, *post hoc* analysis was performed for each level of a corresponding variable.

For the LES treated as continuous variable, analytic procedures are described in Supplementary material S2.4.

Additionally, this study was the first to examine the interaction between IL-6-related SNPs and psychosocial stressors on IL-6 diurnal index, and therefore *a priori* sample size estimation could not be performed. However, for a confirmatory purpose, we performed a *post hoc* power analysis using G*Power 3 (88), based on the effect that was observed to be significant in this study.

#### 2.9.3. Path analysis on the relationship between IL-6 secretion, amygdala emotional reactivity, and depressive/anxiety symptoms under gene-stressor interactions

Focusing on the variables found to be significant in the above-mentioned analyses, we performed path analysis to comprehensively evaluate the pathways from amygdala emotional reactivity to IL-6 secretion and depressive/anxiety symptoms under gene-stressor interactions. A gene-stressor interaction affecting IL-6 levels was calculated by multiplying the NLC group (i.e., NLC+, 1; or NLC−, 0) and SNP type (e.g., for rs1800796, C/C type, 1; or C/G + G/G type, 0), and included in the model for DNA subsample. Standardized *β* values were calculated as each path coefficient. The results of path analysis in the whole sample (i.e., excluding the gene-stressor interaction) are also reported.

#### 2.9.4. IL-6-related brain functional connectivity

To explore any FC associated with the IL-6 index throughout the brain, we performed a correlation analysis using the CONN default atlas (i.e., comprised of FSL Harvard-Oxford atlas^6^ and Automated Anatomical Labeling atlas^7^), covering the whole brain. For the amygdala only, we employed the Jüelich histological atlas, instead of the CONN atlas, to define its subnuclei (i.e., BLA and CEM on both sides). Thus, the total number of regions was 134, including the amygdala subnuclei, hippocampus, and PFC. The time series of BOLD signals were averaged across all voxels within each region, and FC values between the regions were calculated bivariately. False-discovery rate (FDR) correction was applied for multiple tests.

For all analyses, the effects of age, sex, BMI, and sleep duration were controlled since they were identified as IL-6 index confounders (see Supplementary Tables S1,S2; Supplementary material S2.1). Additionally, effects of the time interval between MRI scans and saliva collection and handedness, defined by the Edinburgh inventory (89), were controlled for in fMRI-related analysis. All analyses, except the whole-brain activity and FC analyses, were performed with SPSS version 27.0 J.^8^ The statistical threshold was set at *p* < 0.05 (2-sided) unless otherwise stated.

## 3. Results

### 3.1. Participants

Of the 123 enrolled participants, two did not provide saliva samples and one had an MRI data writing error. For the IL-6 assay, the data whose absorbance values could not be measured with an absorptiometer were excluded from the analysis. Consequently, 1,119 (93.7%) of the total 1,200 IL-6 measurements (i.e., the remaining 120 individuals with 10 measurements each) were included. When IL-6 data was available for only one of the 2 days (i.e., data missing from any time point for either day), the two-day average data was substituted by this single-day data. Of the 120 participants, 12 were excluded from the entire analysis because their IL-6 data were not available for one or more corresponding time points for both days. Thus, we analyzed the data of 108 participants as a final data set (Table 1). Of these participants, two women regularly took oral contraceptives and none were engaged in shift work. Although no participant met the criteria for a current MDD episode, 12 of them had significant depression levels defined by the BDI-II cut-off score, indicating that they were at a high-risk state for MDD. These individuals had moderate levels of depression (BDI-II: Mean ± SD = 22.6 ± 8.5), and were not significantly different from non-depressive individuals in terms of age and sex (age: *t* = 0.34, *p* = 0.74, Mean ± SD = 26.8 ± 11.9 and 27.9 ± 11.4, respectively and sex: *χ^2^* = 0.68, *p* = 0.54, 66.7% and 54.2%, respectively, for female ratios).

**Table 1:** Descriptive statistics of participants’ characteristics and psychological and biological data.

Characteristic	Mean	SD
Age	27.8	11.4
Sex (% of females)	55.6	
Handedness (% with right-handedness)	93.5	
Smoking (% with no smoking habit)	96.3	
BMI	20.9	2.9
Daily caffeine intake (mg)	87.3	68.4
Monthly alcohol consumption (unit)	19.1	32.3
Years of education	15.2	1.7
Days between MRI and saliva collection	4.3	3.9
BDI-II		
Depressive symptom score	7.5	7.0
HSCL		
Anxiety symptom score	10.2	3.2
Depressive symptom score	18.2	5.2
LES		
Negative impact score	4.1	4.9
Positive impact score	3.5	4.1
Balanced impact score	0.6	5.8
SNP^a^		
*IL6* (rs1800796) (%)		
C/C	56.2	
C/G and G/G	43.8	
*IL6R* (rs2228145) (%)		
C/C	64.4	
C/A and A/A	35.6	
IL-6 (pg/ml)		
Diurnal index (Time 5 - Time 4)	6.5	9.5
Total output (AUC_g_)	190.9	146.1
Time 1 (at awakening)	14.3	11.8
Time 2 (30 min after awakening)	13.7	13.8
Time 3 (midday)	10.4	9.5
Time 4 (evening)	9.6	8.5
Time 5 (bedtime)	16.1	13.0

*n* = 108. ^a^SNP data is based on *n* = 73. For IL-6, raw values (winsorized) are described. Saliva collection time at T1, T2, T3, T4, and T5 was as follows: 7:09 (± 1:16), 7:46 (± 1:17), 12:06 (± 0:20), 18:03 (± 0:19), and 0:20 (± 1:09), respectively. BMI, body mass index; BDI-II, Beck Depression Inventory-II; HSCL, Hopkins Symptom Checklist; LES, Life Experiences Survey; SNP, single-nucleotide polymorphism; IL-6, interleukin-6; IL-6R, interleukin-6 receptor; AUCg, area under the curve with respect to ground.

**Figure 3:**
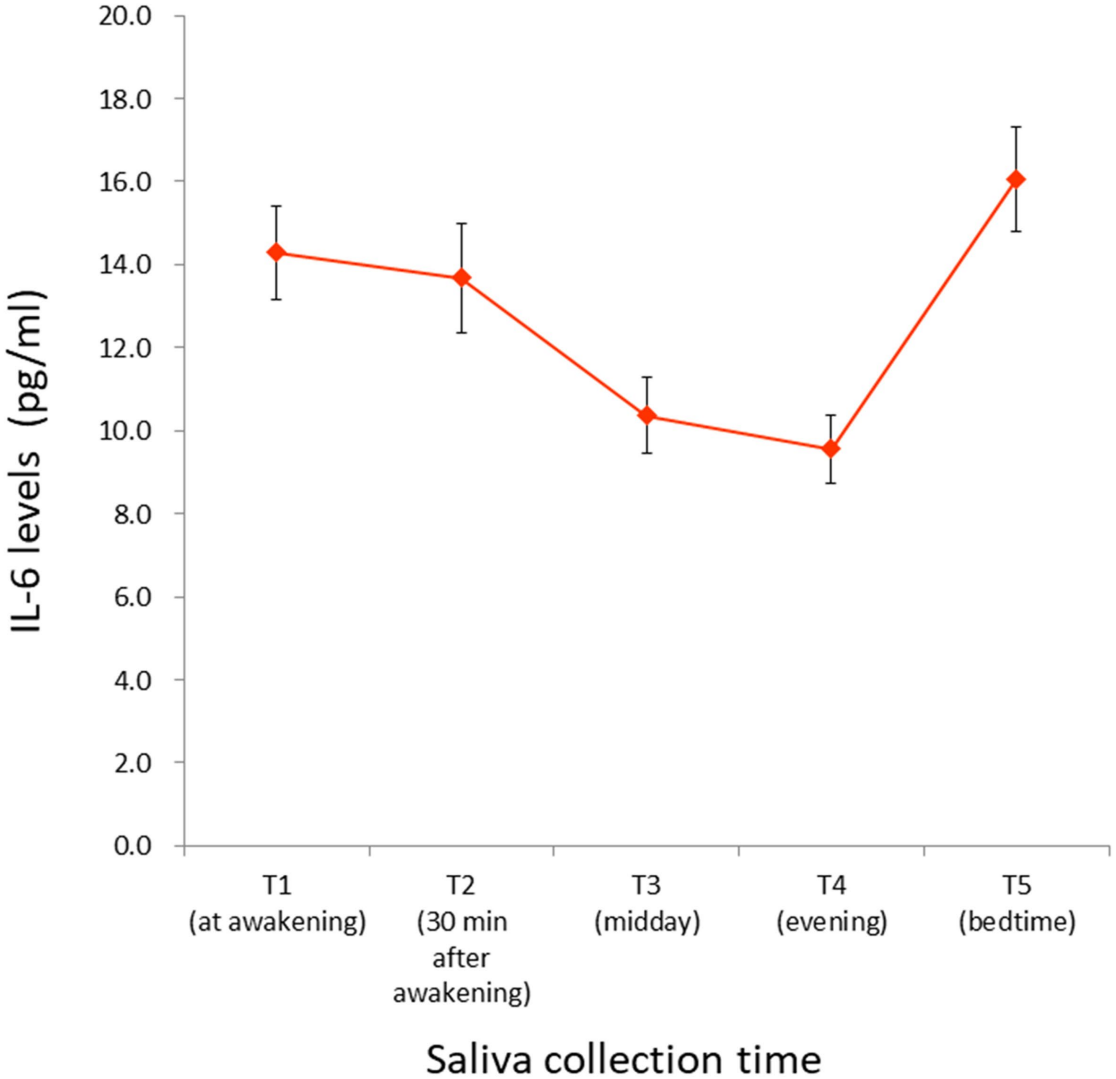
IL-6 levels measured at each time point. Raw values (winsorized) are illustrated. Saliva collection time at T1, T2, T3, T4, and T5 was as follows: 7:09 (± 1:16), 7:46 (± 1:17), 12:06 (± 0:20), 18:03 (± 0:19), and 0:20 (± 1:09), respectively. For the numeric values, see Table 1. Bars indicate the standard error of the means. IL-6, interleukin-6.

Saliva collection time at T1, T2, T3, T4, and T5 was as follows: 7:09 (± 1:16), 7:46 (± 1:17), 12:06 (± 0:20), 18:03 (± 0:19), and 0:20 (± 1:09), respectively. Average and standard errors of raw IL-6 values at each time point are shown in Table 1 and Figure 3. The observed IL-6 diurnal pattern was similar to that reported in our previous study (46), confirming that the salivary IL-6 levels were properly measured in this study. Since IL-6 diurnal index and total output did not follow a normal distribution, they were square-root transformed (*W* = 0.65, *p* < 0.001; *W* = 0.71, *p* < 0.001, respectively).

Likewise, the balanced impact score of LES did not follow a normal distribution (*W* = 0.95, *p* < 0.001), and thus participants were classified into those who experienced NLC and those who did not.

For fMRI data, 11 participants were unable to respond by the time the next screen was presented in more than 30% of the total trials since the timing of stimulus presentation was synchronized with that of each MR signal. The overall hit ratio was 85.3 ± 11.4% in the 97 participants, indicating that they concentrated well on the task. In addition, the time of fMRI scans was 13:39 (± 1:22). The scanning time did not affect amygdala activity (see Supplementary material S2.3).

Furthermore, there was no significant difference between the whole sample (*n* = 108) and the DNA subsample (*n* = 73) in age, sex, IL-6 diurnal index, or total IL-6 output (*t* = 1.10, *df* = 179, *p* = 0.27; *X^2^* (1, 181) = 4.2, *p* = 0.55; *t* = 0.58, *df* = 179, *p* = 0.57; *t* = −0.36, *df* = 179, *p* = 0.72; respectively).

### 3.2. Relationships between IL-6 levels, amygdala emotional reactivity, and depressive/anxiety symptoms

**Figure 4:**
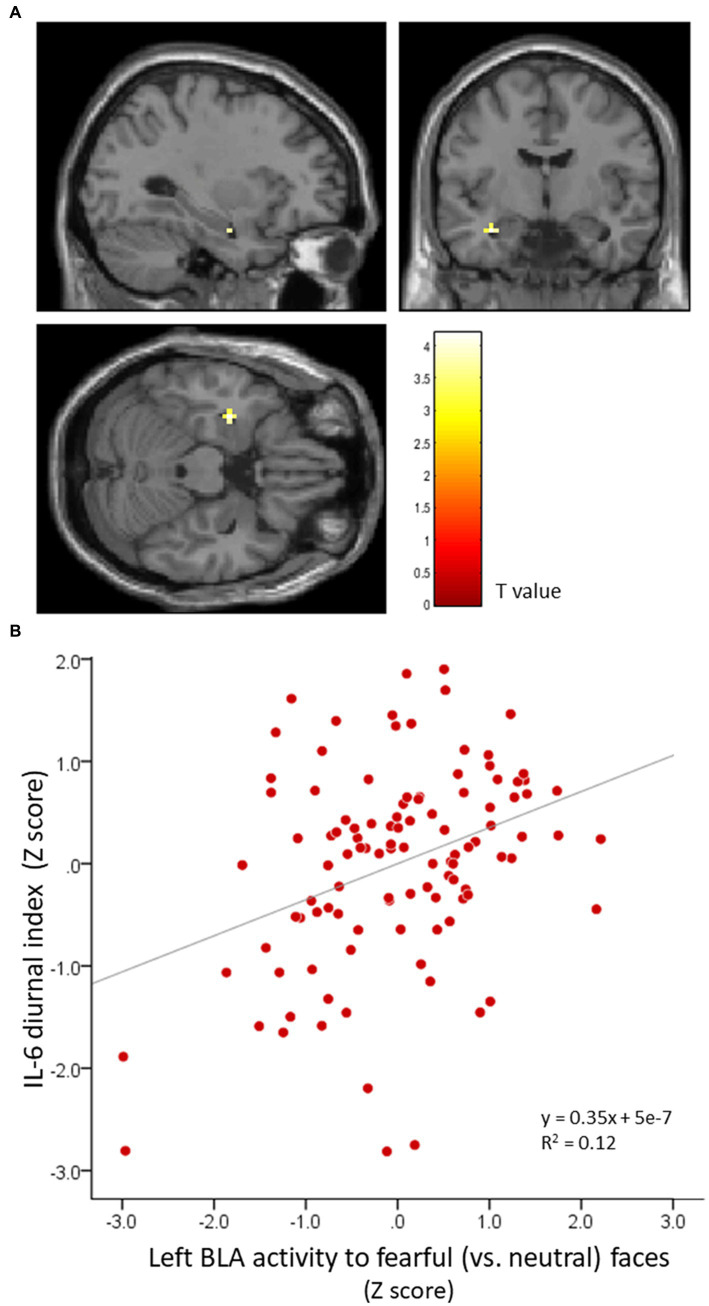
BLA activity in response to fearful (vs. neutral) faces is significantly correlated with the IL-6 diurnal index. Effects of age, sex, BMI, sleep duration, handedness, and time interval from MRI scans to saliva collection were controlled for. **(A)** Significant peak cluster correlated with the IL-6 diurnal index. **(B)** Scatter plot on the relationship between the IL-6 diurnal index and left BLA activity. BLA, basolateral amygdala; IL-6, interleukin-6; BMI, body mass index.

Whole-brain analysis revealed that IL-6 diurnal index had a significantly positive association with left BLA activity to fearful (vs. neutral) faces (MNI: −30 −7 −22, *t* = 3.67, FWE-corrected *p* = 0.003, 54 mm^3^; Figure 4), such that the more the IL-6 diurnal index diminished, the more the BLA activity decreased. No other significant cluster was found in the right BLA, CEM, and other brain regions. For IL-6 total output, no significant correlation was found with activity in any brain region.

Additional correlation analysis showed that the IL-6 diurnal index was associated with BDI-II depressive symptoms only, with increased blunting of the IL-6 diurnal pattern corresponding with greater depressive symptoms, although this correlation failed to reach the Bonferroni-corrected statistical threshold (*r* = −0.24, *p* = 0.018). The IL-6 diurnal index did not correlate with HSCL anxiety and depressive symptoms (Table 2). Despite the distinct correlation with the IL-6 diurnal index (*r* = 0.30, *p* = 0.002) as observed in the whole-brain analysis, the left BLA activity was uncorrelated with BDI-II depressive symptoms (*r* = 0.04, *p* = 0.67), although it had a relative correlation with HSCL anxiety symptoms (*r* = 0.20, *p* = 0.04).

**Table 2:** Correlations between IL-6 indices, amygdala subnuclei activity to fearful (vs. neutral) faces, and stress-related mental symptoms.

Variable	IL-6 diurnal rhythm index	Total IL-6 output	L BLA	R BLA	L CEM	R CEM	L HC	R HC	Depression (BDI-II)	Anxiety (HSCL)	Depression (HSCL)
IL-6 diurnal rhythm index	–										
IL-6 total output	0.15	–									
L BLA	**0.30** ^b^	0.04	–								
R BLA	0.10	0.10	**0.56** ^a^	–							
L CEM	0.25^c^	−0.02	**0.65** ^a^	**0.45** ^a^	–						
R CEM	0.02	0.07	**0.46** ^a^	**0.56** ^a^	**0.57** ^a^	–					
L HC	0.27^b^	−0.01	**0.52** ^a^	**0.43** ^a^	**0.64** ^a^	**0.45** ^a^	–				
R HC	0.12	0.09	**0.47** ^a^	**0.52** ^a^	**0.54** ^a^	**0.42** ^a^		–			
Depression (BDI-II)	−0.24^c^	−0.00	0.04	0.08	−0.01	0.03	0.09	0.18	–		
Anxiety (HSCL)	−0.14	−0.02	0.20^c^	0.19	0.15	0.15	0.17	0.25^c^	**0.71** ^a^	–	
Depression (HSCL)	−0.14	−0.00	0.08	0.18	0.03	0.06	0.07	0.18	**0.78** ^a^	**0.80** ^a^	–

*^a^p* < 0.001, ^b^*p* < 0.01, ^c^*p* < 0.05. The statistical threshold was corrected with Bonferroni correction based on the number of tested variables.

Values that survived Bonferroni correction (*p* < 0.0045) are shown in bold.

Effects of age, sex, BMI, sleep duration, handedness, and time interval between MRI and saliva collection were adjusted for in the analysis.

Values of BLA and CEM are those extracted from the masks defined by Jüelich histological atlas (i.e., pre-defined, whole structural mask); and for those of HC, by FSL Harvard-Oxford atlas (http://www.cma.mgh.harvard.edu/fslatlas.html).

IL-6, interleukin-6; L, left; R, right; BLA, basolateral amygdala; CEM, centromedial amygdala; HC, hippocampus; BMI, body mass index; HSCL, Hopkins Symptom Checklist; BDI-II, Beck Depression Inventory-II.

**Figure 5:**
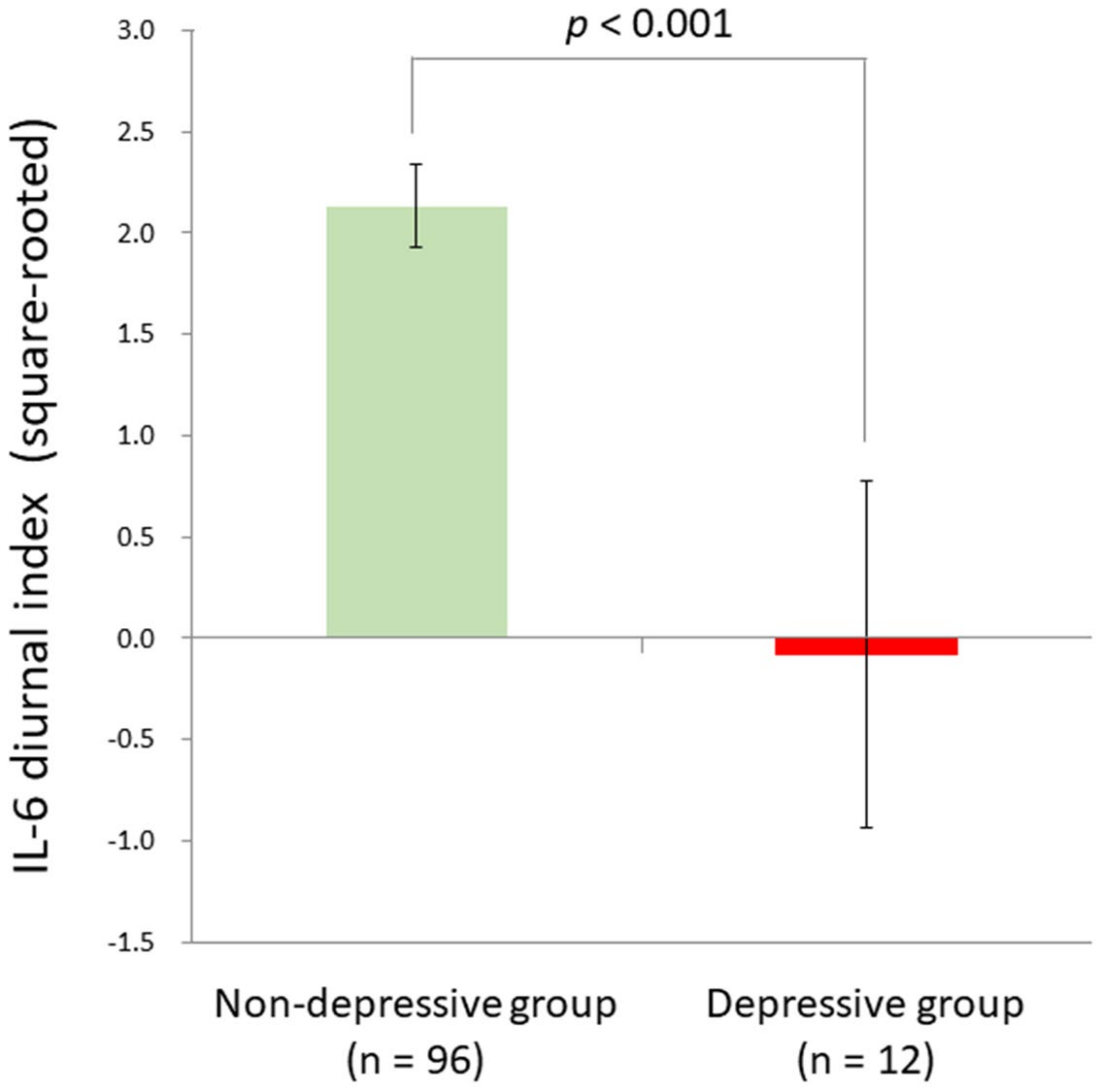
Significant group difference between depressive and non-depressive individuals in IL-6 diurnal secretion. Effects of age, sex, BMI, and sleep duration were controlled for. The depressive group had a significantly blunted IL-6 diurnal pattern (mean ± SD = −0.08 ± 2.98) as compared to the non-depressive group (mean ± SD = 2.13 ± 2.00) [*F* (1,102) = 14.8, *p* < 0.001, partial *η*^2^ = 0.13]. IL-6, interleukin-6; BMI, body mass index.

Although the linear relationship between BDI-II depression and IL-6 diurnal index slightly failed to survive the Bonferroni correction, we also compared depressive individuals defined by the BDI-II cut-off score (*n* = 12) and non-depressive ones (*n* = 96) with ANCOVA controlling for age, sex, sleep duration, and BMI, in consideration of a possible non-linearity of biomarkers as suggested in the allostatic load model (1). As a result, we found a significant difference between the two groups (*F*[1,102] = 14.8, *p* < 0.001, partial *η*^2^ = 0.13). The depressive group had a significantly blunted IL-6 diurnal pattern (mean ± SD = −0.08 ± 2.98) as compared to the non-depressive group (mean ± SD = 2.13 ± 2.00; Figure 5).

### 3.3. Effects of gene-stressor interactions on IL-6 secretion

Three-way ANCOVA demonstrated a significant rs1800796 × NLC interaction effect [*F*(1,61) = 15.1, *p* < 0.001, partial *η*^2^ = 0.20], with a significant main effect detected for NLC [*F*(1,61) = 4.26, *p* = 0.04, partial *η*^2^ = 0.07]. No other significant effects were observed: *F* (1,61) = 0.68, *p* = 0.41 for *IL6*; *F*(1,61) = 0.44, *p* = 0.51 for *IL6R*; *F*(1,61) = 0.00, *p* = 0.99 for NLC × *IL6R*; *F*(1,61) = 0.05, *p* = 0.82 for *IL6* × *IL6R*; *F*(1,61) = 0.69, *p* = 0.41 for NLC × *IL6* × *IL6R*.

**Figure 6:**
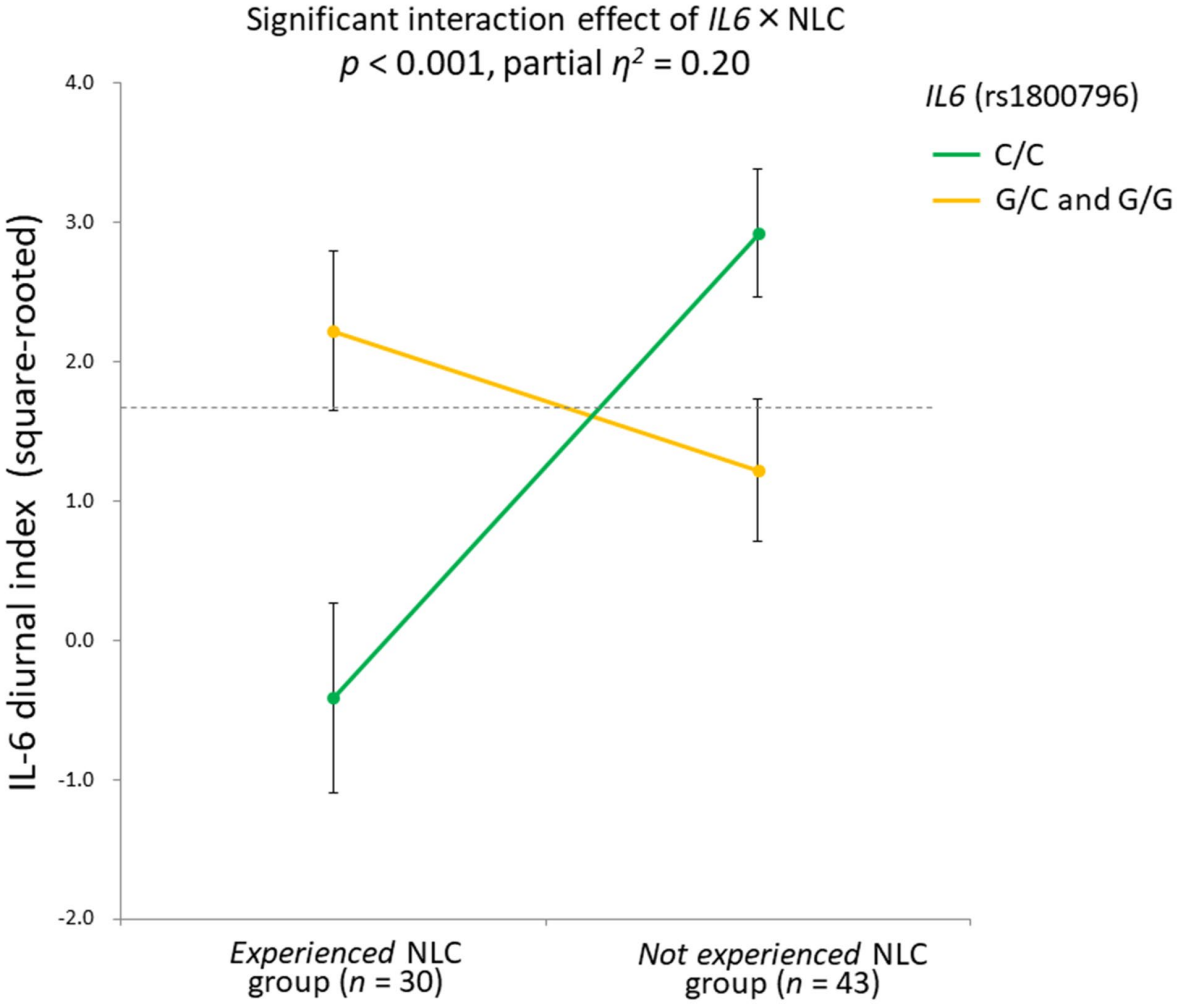
Significant gene-stressor interaction impacts IL-6 diurnal secretion. The dotted line indicates the mean values of the IL-6 diurnal index (square-rooted): 1.69 ± 2.42. Error bars indicate the standard error of the means. Effects of age, sex, BMI, and sleep duration were controlled for. Significant difference found in the C/C group [*n* = 41, *F*(1,35) = 19.71, *p* < 0.001], but not in the G/C + G/G group [*n* = 32, *F*(1,26) = 1.22, *p* = 0.28]. In the C/C group, the IL-6 diurnal index significantly diminished under the presence of NLC (*n* = 16, mean ± SD = −0.32 ± 3.22, -0.80 ± 1.11 in *Z* score) but not under its absence (*n* = 25, mean ± SD = 2.80 ± 1.56, 0.22 ± 0.81 in Z score). IL-6, interleukin-6; BMI, body mass index; NLC, negative life change.

When the significant rs1800796 × NLC effect was parsed into a simple main effect, a significant difference was found in the C/C group [*n* = 41, *F*(1,35) = 19.71, *p* < 0.001], but not in the G/C + G/G group [*n* = 32, *F*(1,26) = 1.22, *p* = 0.28]. Specifically, in the C/C group, the IL-6 diurnal index was significantly diminished under the presence of NLC (*n* = 16, mean ± SD = −0.32 ± 3.22, −0.80 ± 1.11 in Z score) but not under its absence (*n* = 25, mean ± SD = 2.80 ± 1.56, 0.22 ± 0.81 in Z score; Figure 6).

Additionally, our sample size for DNA analysis was observed to have a power of 0.99 to detect the observed effect size for the *IL6* × NLC interaction (i.e., partial *η*^2^ = 0.20) at an *α* = 0.05.

Further, a multiple regression analysis where the LES balanced impact score was included as a continuous variable similarly found the rs1800796 × LES interaction effect (*β* = 0.82, *t* = 2.66, *p* = 0.010; see Supplementary material S2.4), confirming the *IL6* SNP-psychosocial stressor interaction effect on the IL-6 diurnal index.

### 3.4. Path analysis on the relationship between IL-6 diurnal index, BLA activity, and depressive symptoms under gene-stressor interaction

Since the correlation of IL-6 diurnal index with BDI-II depression was still relatively significant, we proceeded to an exploratory path analysis on possible pathways from BLA activity to the IL-6 diurnal index and BDI-II depression under the rs1800796 × NLC interaction.

**Figure 7:**
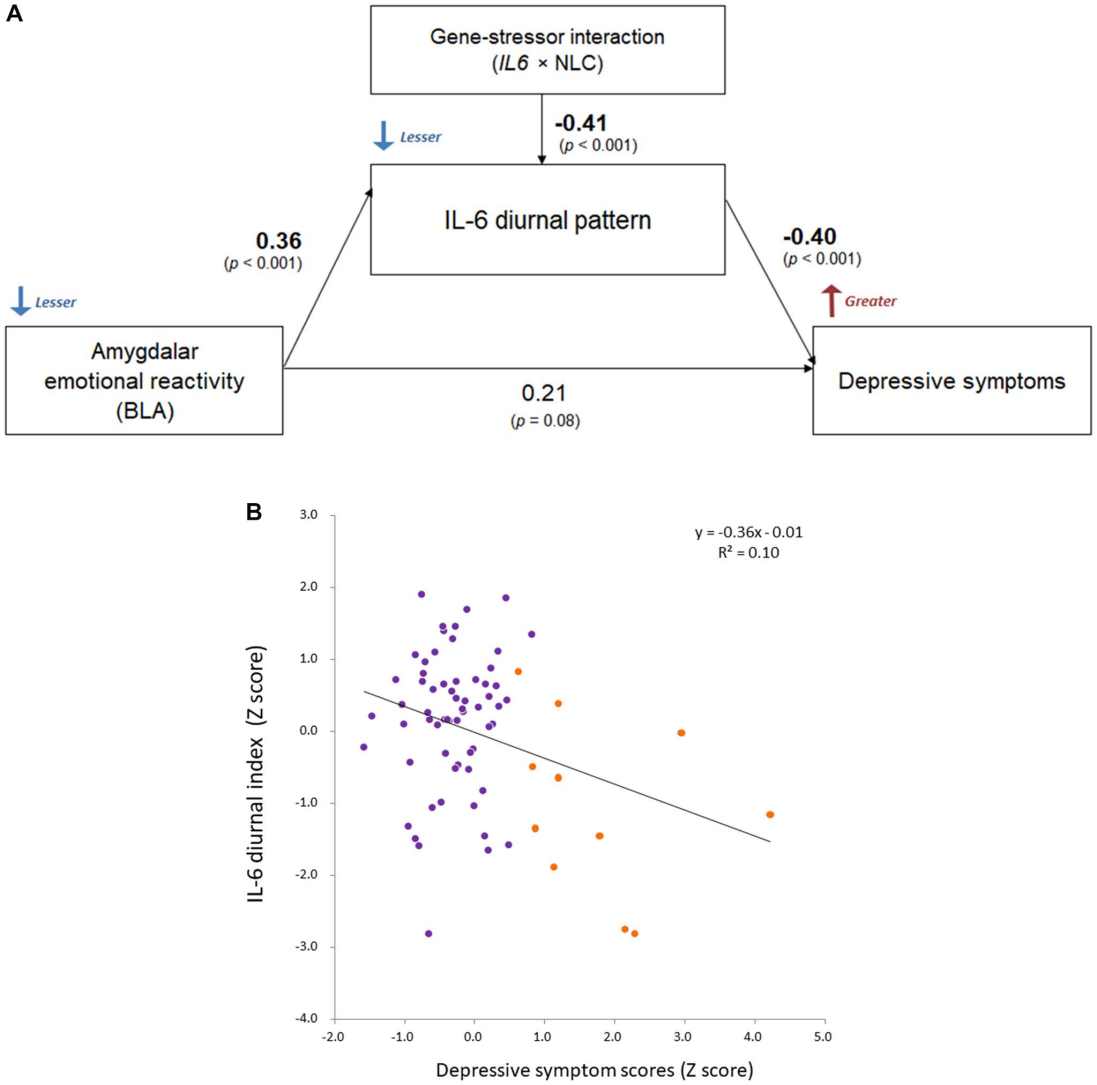
Comprehensive relationship between IL-6 diurnal index, BLA activity, and depression under gene-stressor interaction. Effects of age, sex, BMI, sleep duration, handedness, and time interval from MRI scans to saliva collection were controlled for. The values of left BLA activity were extracted based on the anatomical definition of Jüelich histological atlas. Depressive symptoms were assessed with BDI-II. **(A)** Results of path analysis (*n* = 73). Standardized *β* values are shown as each path coefficient. **(B)** Scatter plot on the relationship between the IL-6 diurnal index and depressive symptoms. Dots shown in orange color indicate individuals with significant levels of depression (i.e., scores ≥14 on BDI-II; *n* = 11 in DNA sample). BLA, basolateral amygdala; IL-6, interleukin-6; BMI, body mass index; *IL6*, rs1800796 single-nucleotide polymorphism; NLC, negative life change; BDI-II, Beck Depression Inventory-II.

Based on the significant rs1800796 × NLC effect on IL-6 diurnal index and the relative correlation, Path analysis demonstrated that the diminished IL-6 diurnal index specifically explained greater depressive symptoms (*β* = −0.40, *p* < 0.001; respectively), being affected by the left BLA activity and the rs1800796 × NLC interaction (*β* = 0.36, *p* < 0.001; *β* = −0.41, *p* < 0.001; respectively; Figure 7).

Additionally, the path coefficients in the DNA subsample were comparable to those in the whole sample where the gene-stressor interaction was not included (BLA–IL-6: *β* = 0.29, *p* = 0.002; IL-6–depression: *β* = −0.28, *p* = 0.004; BLA–depression: *β* = 0.16, *p* = 0.10), supporting the observed relationships.

### 3.5. IL-6-related brain functional connectivity

**Figure 8:**
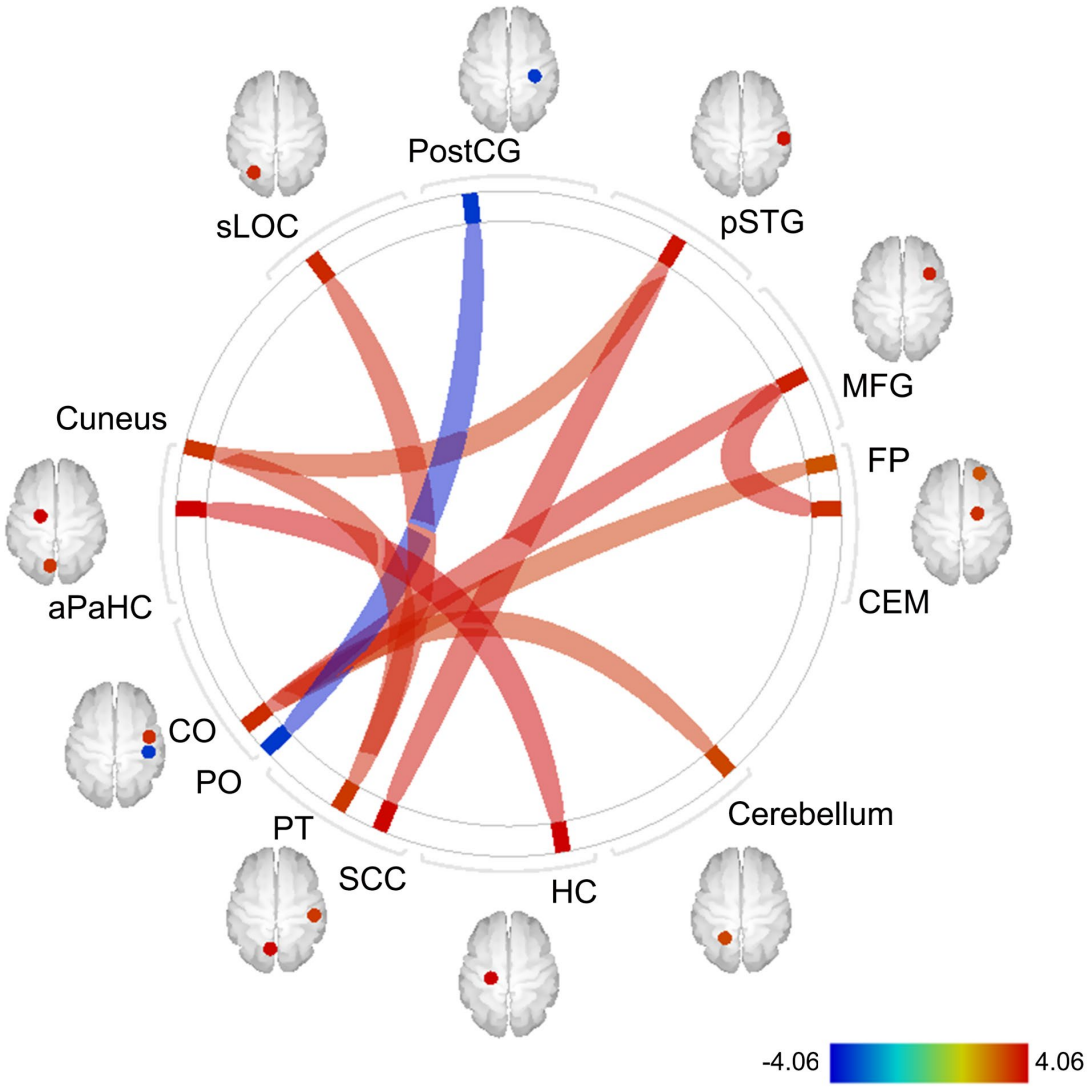
Exploratory whole-brain ROI-based FC analysis: correlations with the IL-6 diurnal index. Controlled for age, sex, BMI, sleep duration, handedness, and the time interval between MRI scans and saliva collection. The color bar indicates *t* values. Red and blue lines indicate increased and decreased FC, respectively. The width of lines is proportionate to statistical significance values. ROI, region of interest; FC, functional connectivity; IL-6, interleukin-6; BMI, body mass index; postCG, postcentral gyrus; sLOC, superior lateral occipital cortex; aPaHC, anterior parahippocampal gyrus; CO, central operculum cortex; PO, parietal operculum cortex; PT, planum temporale; SCC, supracalcarine cortex; HC, hippocampus; CEM, centromedial amygdala; MFG, middle frontal gyrus; pSTG, posterior division of superior temporal gyrus; FP, frontal pole.

Whole-brain FC analysis revealed that the IL-6 diurnal index was significantly associated with increased FC between the CEM and middle frontal gyrus (MFG) on the right side (*t* = 3.92, FDR-corrected *p* = 0.012), as well as other cortical regions (Figure 8). All the significant FC values are reported in Table 3.

**Table 3:** The results of whole-brain FC analysis: correlations with IL-6 diurnal index.

Seed region		Target region		*t*-value	value of *p* (FDR-corrected)
R	Middle frontal gyrus	R	Centromedial nucleus of the amygdala	3.92	0.012
		R	Central operculum cortex	3.90	0.012
R	Central operculum cortex	L	Cerebellum	3.45	0.049
		R	Frontal pole	3.36	0.049
L	Parahippocampal gyrus (anterior division)	L	Hippocampus	4.06	0.013
R	Planum temporale	L	Lateral occipital cortex (superior division)	3.72	0.026
		L	Cuneal cortex	3.67	0.026
R	Superior temporal gyrus (posterior division)			3.48	0.049
		L	Supracalcarine cortex	3.93	0.021
R	Postcentral gyrus	R	Parietal operculum cortex	−3.78	0.035

Effects of age, sex, BMI, sleep duration, handedness, and days between MRI and saliva collection were controlled. The CONN default atlas and Jüelich histological atlas (only for BLA and CEM on both sides) were used for defining each brain region.

IL-6, interleukin-6; ROI, region of interest; FC, functional connectivity; L, left; R, right.

To summarize, the diminished IL-6 diurnal pattern was associated with the hypoactivation of the left BLA to fearful (vs. neutral) faces, and was predominantly observed in individuals with the C-allele homozygotes of *IL6* and NLC in the past year. When considered comprehensively, the blunted diurnal pattern predicted greater depressive symptoms, modulated by the amygdala hypoactivity and rs1800796-NLC interactions.

## 4. Discussion

The current study investigated the relationship between IL-6 levels, amygdala emotional reactivity, and depression/anxiety, considering gene-psychosocial stressor interactions in nonclinical participants with various levels of depression/anxiety. We specifically hypothesized that disrupted IL-6 secretion would be associated with perturbed amygdala emotional reactivity and greater depressive/anxiety symptoms; and that it would be predicted by the interactions between psychosocial stressors and *IL6*/*IL6R*.

In support of our hypotheses, we demonstrated that a diminished IL-6 diurnal index was associated with decreased amygdala activity, especially in the BLA, in response to fearful (vs. neutral) faces, as well as greater depressive symptoms, through a comprehensive examination. The interaction between NLC and the *IL6* SNP explained the diminished IL-6 diurnal pattern. The current study provides evidence on the relationship between IL-6 secretion, amygdala emotional reactivity, and depression, indicating that disrupted IL-6 secretion could affect greater depressive symptoms, being modulated by amygdala hyporeactivity and gene-psychosocial stressor interactions.

Specifically, the IL-6 diurnal pattern, but not the total IL-6 output, was associated with BLA response to emotional stimuli. The lack of association between total IL-6 output and amygdala activity is partially consistent with several previous findings (56–59). First, this could potentially be because the IL-6 diurnal index and amygdala emotional responses capture a dynamic facet of reactivity rather than a static component. Moreover, while the IL-6 diurnal index was associated with depressive symptoms, the total IL-6 output did not correlate with anxiety or depressive symptoms. This was possibly because the participants involved did not have psychiatric diagnoses, suggesting the diurnal pattern of IL-6, rather than the total output, to be a more sensitive measure to the link between the immune system and mental health problems in the general population.

Importantly, the blunting of the IL-6 diurnal pattern was associated with both decreased amygdala responses to fearful faces and greater depressive symptoms. These findings are partly consistent with previous meta-analyses on the weakened emotional responses of the dorsal and lateral amygdala in PTSD (25) and MDD (23), as well as with previous findings on a flattened IL-6 diurnal pattern in individuals who underwent childhood trauma or combat-zone deployment (54, 55), which confer a risk of developing stress-related mental disorders (90, 91). The hypoactivation or lack of activation of the amygdala toward emotional stimuli has been implicated in both emotional and autonomic blunting (25, 92). The blunting of IL-6 diurnal secretion might be associated with the declined regulatory function of stress-related cytokines’ expression (18, 19) and weakened emotional reactivity (25, 92) in the amygdala, which potentially increases vulnerability to depression characterized by blunted affect such as anhedonia and learned helplessness (93). These findings may lead to the development of biomarkers for the early detection of depressive disorders, although the causal relationships between the variables are subject to scrutiny.

The amygdala regulates stress-related inflammatory cytokine expression via the norepinephrine system (18, 94). The BLA rapidly processes a potential threat, while the CEM elicits emotional responses, including norepinephrine secretion. More specifically, the CEM, where alpha adrenoreceptors are richly expressed (95, 96), receives catecholaminergic innervation from the locus coeruleus (97). Despite tight connections between BLA, CEM, and adjacent hippocampus (98), the activity of the BLA survived the correction for multiple testing (Table 2). This might have been because the BLA is reported to be susceptible to the effects of stress, such as dendritic remodeling, as mentioned earlier (6). In addition, this is also potentially due to the amygdala response elicited by the current emotional task being more relevant to the left BLA (84). Indeed, our previous study observed distinct activity in the amygdala, including the BLA on the left side (69), supporting the notion that this task is particularly sensitive to the left BLA. Although a consensus has not been reached on functional lateralization of the amygdala, some evidence suggests that the left side might be more strongly involved in emotion than the right (99). Moreover, although the Jüelich histological atlas is a reliable source in amygdala dissection, given the task-dependent activity and limit of fMRI spatial resolution, the CEM and hippocampus may also impact the IL-6 diurnal pattern, warranting further investigation.

Indeed, FC analysis demonstrated a significant positive association between the IL-6 diurnal pattern and FC between CEM and MFG, largely corresponding to the dorsolateral PFC (DLPFC), in response to fearful (vs. neutral) faces. Specifically, the more the IL-6 diurnal pattern diminished, the more this FC weakened. For the DLPFC, a previous positron emission tomography study reported a positive correlation between immune marker levels, including natural killer and helper T cell levels, and regional cerebral blood flow during a mental arithmetic task with low controllability (100). Moreover, an fMRI study revealed that IL-6 levels were negatively correlated with MFG activity, which decreased depending on an increase in cognitive load during the N-back task (101). Therefore, CEM-DLPFC connectivity might affect the secretion of immune/inflammatory markers according to cognitive load and stress controllability. Given the fMRI temporal resolution, the causal relationship between BLA activity and CEM-related FC remains unclear. Nevertheless, CEM connectivity might impact IL-6 diurnal rhythm modulation, given norepinephrine’s immune regulatory role (19). However, this is only a speculation that requires longitudinal investigation.

Furthermore, we found that the *IL6* SNP-psychosocial stressor interaction predicted a diminished IL-6 diurnal pattern. Notably, individuals with the C/C genotype exhibited distinctively weakened (approaching −1 SD) patterns when experiencing NLC in the past year, whereas those with C/G and G/G genotypes did not. These findings suggest that diurnal IL-6 secretion may be influenced by gene-stressor interactions, supporting previous research (37, 38). Although future research needs to elucidate the exact mechanism, the blunting of IL-6 diurnal secretion might be possibly due to excessive stress (54), as the blunting of glucocorticoid secretion has been similarly observed after extreme stress (102). Given that the C substitution of rs1800796 has been reported to be associated with lower IL-6 levels (40, 43), individuals with the C/C genotype might be vulnerable to the disrupted regulation of IL-6 when exposed to stressors as compared to G-allele carriers. The effect of IL6-stressor interactions on the amygdala was not shown in this study, but the expression of IL-6 might be dampened in the amygdala, which might partially contribute to the blunted affect of depression (94). The diathesis-stress model for the development of MDD (103) partly accords with this possibility, and the disruption of the IL-6 diurnal rhythm, influenced by the individual’s genetic predispositions and the intensity of environmental stress, might be a key predictor for depression. However, this speculation needs to be thoroughly investigated in future research.

### 4.1. Limitations and future directions

Several limitations should be considered when interpreting these results. First, our sample for the SNP-related analysis was not very large, although the sample size was sufficiently powered to detect the observed significant interaction effect according to *post hoc* power analysis. Still, future research with larger sample sizes is necessary. Second, information on oral hygiene of the participants was unavailable in this study. However, even if the IL-6 levels at each time point were affected by inflammation due to oral hygiene, it would not significantly undermine the quality of IL-6 diurnal index data as it was calculated based on differences between two-time points, conceivably eliminating the systematic error due to such inflammation. In future research, information on oral hygiene should be obtained. Third, this was a cross-sectional study and did not reveal potential causal relationships between IL-6 levels, amygdala emotional reactivity, and depressive symptoms. Future research should elucidate these causal relationships by simultaneously performing fMRI and other imaging techniques with a higher temporal resolution, such as electroencephalography. Furthermore, the development of indices to assess individuals’ regulatory functions of the immune system beyond acute inflammation (i.e., not only phasic but also tonic changes of the immune system) might enable the early detection of high-risk individuals whose psychopathology is yet to be manifested and the effective prevention for them by ameliorating their immune system dysregulation. To capture such baseline regulatory functions, consecutive measurements of unstimulated biological markers on a daily basis, such as IL-6 diurnal patterns measured in this study and spontaneous low-frequency fluctuations measured by resting-state fMRI, might bring a new insight to psychiatry research.

### 4.2. Conclusion

We showed that blunting of the IL-6 diurnal pattern was associated with both amygdala (especially BLA) hypoactivity in response to fearful (vs. neutral) faces and greater depressive symptoms in a sizeable community sample with various levels of depression. Disrupted IL-6 diurnal rhythm was explained by the interaction between *IL6* polymorphism and stressful life events. These findings represent the first evidence that diminished IL-6 diurnal rhythm could affect depression, modulated by amygdala emotional hyporeactivity and the interaction between the individual’s genetic predisposition and the intensity of environmental stress. Developing an understanding of this blunted diurnal IL-6 rhythm, a possible precipitant in stress-related mental disorders such as MDD, is expected to facilitate their early detection, prevention, and treatment such as pharmacotherapy to ameliorate the disrupted secretion of inflammatory markers.

## Data availability statement

The original contributions presented in the study are included in the article/Supplementary materials, further inquiries can be directed to the corresponding author.

## Ethics statement

The studies involving human participants were reviewed and approved by the Kitasato University Medical Ethics Organization and the National Center of Neurology and Psychiatry Ethics Committee. The patients/participants provided their written informed consent to participate in this study.

## Author contributions

YH designed the study, wrote the protocol, conducted the psychological assessment and statistical analyses, and wrote the first draft of the manuscript. HH conducted DNA-related statistical analyses and revised the first draft of the manuscript. SM conducted the MRI scans. SI conducted the IL-6 assay. FY performed the IL-6 assay and SNP genotyping. YM and TH supervised the fMRI analyses. YI and HT provided support during the recruitment of participants and assessment. All the authors made critical revisions to the intellectual content of the manuscript and approved the final manuscript.

## Funding

The authors received a Grant-in-Aid for Scientific Research (B) (Nos. 18H01094 and 22H01092 to YH) and a Grant-in-Aid for Scientific Research (C) (No. 20 K07937 to HH) from the Japanese Society for the Promotion of Science, research grants from the Uehara Memorial Foundation (to YH), the Suzuken Memorial Foundation, and Terumo Life Science Foundation (to HH). Funding sources did not influence any aspect of the work, including design, data collection, analyses, interpretation, writing, or submission, detailed in the current manuscript.

## Conflict of interest

The authors declare that the research was conducted in the absence of any commercial or financial relationships that could be construed as a potential conflict of interest.

## Publisher’s note

All claims expressed in this article are solely those of the authors and do not necessarily represent those of their affiliated organizations, or those of the publisher, the editors and the reviewers. Any product that may be evaluated in this article, or claim that may be made by its manufacturer, is not guaranteed or endorsed by the publisher.

## Footnotes

^1^ http://www.fil.ion.ucl.ac.uk/spm/software/spm12

^2^ http://www.nitrc.org/projects/conn

^3^ https://www.mathworks.com/

^4^ https://fsl.fmrib.ox.ac.uk/fsl/fslwiki/Atlases/Juelich

^5^ http://marsbar.sourceforge.net/

^6^ http://www.cma.mgh.harvard.edu/fsl atlas.html

^7^ http://www.gin.cnrs.fr/AAL

^8^ https://www.ibm.com/products/spss-statistics
