# Supplementary material for: Blunted diurnal interleukin-6 rhythm is associated with amygdala emotional hyporeactivity and depression: a modulating role of gene-stressor interactions
Source: Front Psychiatry. 2023 May 30;14:1196235. doi: 10.3389/fpsyt.2023.1196235 (PMC10262086; doi:10.3389/fpsyt.2023.1196235)
Supplement: Supplementary file 1 [file Data_Sheet_1.docx]

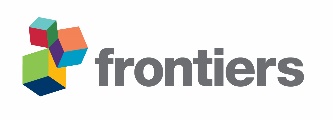
Supplementary Material

Blunted diurnal interleukin-6 rhythm is associated with

amygdala emotional hyporeactivity and depression:

A modulating role of gene-stressor interactions

Yuko Hakamata* Hiroaki Hori, Shinya Mizukami, Shuhei Izawa, Fuyuko Yoshida, Yoshiya Moriguchi, Takashi Hanakawa, Yusuke Inoue, and Hirokuni Tagaya

*** Correspondence:**

**Yuko Hakamata**: [hakamata@med-u.toyama.ac.jp](mailto:hakamata@med-u.toyama.ac.jp)

# Methods

- 1. **S****coring methods for BMI, daily caffeine intake, monthly alcohol consumption, and menstrual period**

***Body mass index (BMI)*:** BMI was calculated using this formula: [weight (kg)]/[height (m)]^2^.

***Daily caffeine intake***: Caffeine intake (mg) was calculated using the following database: https://www.caffeineinformer.com/the-caffeine-database.

***Monthly alcohol consumption***: The formula proposed by the United Kingdom’s National Health Service (https://www.nhs.uk/live-well/alcohol-support/calculating-alcohol-units/) was used to calculate the alcohol units consumed by each participant: [volume consumed (ml)] × [alcohol by volume (%)]/[1000]. Drinking frequency per month (days) was weighted by the unit of alcohol calculated. For example, for a person who drinks a standard glass (175 ml) of wine (12 %) once per week, the monthly alcohol units would be calculated as follows: (175 × 12/1000) × 4 (days per month) = 8.4, whereas for a person who drinks a standard glass of wine twice per week, the alcohol units would be 16.8.

***Menstrual period***: Menstrual status at the start of saliva collection was estimated according to the Ogino method, which has been used for infertility treatment and contraception (1, 2). In this method, the “ovulatory phase” is presumed to occur between two days before and three days after the 14th day before the next anticipated menstruation start date, based on each participant’s typical menstrual period. The periods before and after the ovulatory phase were regarded as the “follicular phase” and “luteal phase,” respectively. Women who menstruated irregularly (e.g., menstruation occurring fortnightly for one month, while later occurring several months after the previous one) and who terminated menstruation were categorized as “irregular cycle” and “menopause,” respectively.

- 1. **Confounder analysis for IL-6**

To consider potential confounders for IL-6, we examined whether total output (AUC_g_) and the diurnal index were significantly associated with the following variables: age, sex, years of education, BMI, daily caffeine intake, smoking habits, monthly alcohol consumption, menstrual period, sleep duration, sleep quality, physical conditions, or perceived stress. The effect of sex was examined using an independent *t*-test, and the menstrual period was analyzed using analysis of variance (ANOVA). Pearson’s correlation analysis was performed for the other quantitative variables.

1. **Results**
   1. **Confounder analysis for IL-6 indices**

The correlations of the IL-6 diurnal index and total output with quantitative variables are reported in **Table S1**. There were significant correlations between IL-6 diurnal index and age (*r* = 0.32, *p* < 0.001) as well as between total IL-6 output and age (*r* = 0.20, *p* = 0.04), BMI (*r* = 0.26, *p* = 0.006), and sleep duration (*r* = -0.20, *p* = 0.04). IL-6 diurnal index also showed a significant difference between the sexes (*t* = -2.17, *df* = 81, *p* = 0.03 for when homoscedasticity was not assumed), such that women (mean = 11.1, SD = 20.7 in raw values) had higher indices than men (mean = 4.7, SD = 14.7 in raw values), while there was no significant difference for total IL-6 output (*t* = 0.07, *df* = 106, *p* = 0.95).

For the menstrual period, no significant differences were found for either of the IL-6 measures: *F* (5, 54) = 0.85, *p* = 0.52 for IL-6 diurnal index, and *F* (5, 54) = 0.48, *p* = 0.79 for total IL-6 output. The mean and SD of these measures in each menstrual group are presented in **Table S2**.

Regarding smoking habits, four participants had a current smoking habit. No significant difference was found in either IL-6 measure between smokers and non-smokers: IL-6 diurnal index, *t* = 2.42, *df* = 106, *p* = 0.81 and IL-6 total output, *t* = -0.06, *df* =106, *p* = 0.95.

Thus, we controlled for the effects of age, sex, BMI, and sleep duration for related analyses.

- 1. **Correlation between potential IL-6 diurnal indices and amygdala subnuclei activity**

For the IL-6 diurnal index, we focused on T5–T4, which reflects an increase in IL-6 level from evening to midnight. For reference, we also calculated correlation coefficients for the other potential diurnal indices with amygdala subnuclei activity in response to fearful (vs. neutral) faces. The results are reported in **Table S3**. The T5–T3 index similarly had a positive correlation with the left BLA activity (*r* = 0.27, *p* = 0.006), although that of T5–T4 was stronger (*r* = 0.31, *p* = 0.002). In contrast, T1–T4 was not significantly correlated with the left BLA activity (*r* = -0.16, *p* = 0.10), indicating that T5–T4 is the most reliable index to capture a typical IL-6 diurnal pattern. Moreover, although these IL-6 diurnal indices were weakly correlated with the left CEM activity (all at *p* < 0.05), the T5–T4 index had the strongest of these correlations (*r* = 0.25, *p* = 0.01).

- 1. **Correlations between fMRI scanning time and amygdala subnuclei activity**

Given the possibility that amygdala activity has a diurnal rhythm (3), correlations between fMRI scanning time and amygdala subnuclei activity were examined. Scanning time was converted as serial values. As a result, no significant correlation was found with any amygdala subnuclei activity in response to emotional stimuli (L BLA: *r* = 0.06, *p* = 0.54; R BLA: *r* = −0.01, *p* = 0.91; L CEM: *r* = 0.10, *p* = 0.32; R CEM: *r* = 0.11, *p* = 0.26).

- 1. **Gene-stressor interaction effect on IL-6 secretion: multiple linear regression analysis with LES balanced impact score as a continuous variable**

As an additional analysis, we performed a multiple linear regression analysis for the IL-6 diurnal pattern as dependent variable with *IL6*, *IL6R*, LES balanced impact score, and the interactions between LES balanced impact score, *IL6*, or *IL6R* as independent variables. Each interaction term was created by multiplying square-root transformed LES score, *IL6* (C/C, 1 and G/C and G/G, 0), or IL6R (C/C, 1 and A/C and A/A, 0), which resulted in the following interaction terms: *IL6*×LES, *IL6R*×LES, *IL6*×*IL6R*, *IL6*×*IL6R*×LES. These interactions, in addition to *IL6*, *IL6R*, and LES balanced impact score, were incorporated into a model with the forced entry method, after the effects of age, sex, BMI, sleep duration, and the time interval between MRI scans and saliva collection were controlled for.

As a result, although the regression model was marginally significant: *F* (12, 72) = 1.89, *adjusted* *R^2^* = 0.13, *p* = 0.055, the interaction effect between *IL6* and LES balanced impact score was significant for IL-6 diurnal index (*β* = −0.82, *t* = 2.66, *p* = 0.010), such that the more individuals with C/C genotype experienced negative life changes, the more their IL-6 diurnal pattern blunted. No other significant predictor was found: *IL6* (*β* = −0.09, *t* = 0.46, *p* = 0.65), *IL6R* (*β* = 0.14, *t* = 0.79, *p* = 0.44), LES (*β* = 0.53, *t* = −1.82, *p* = 0.07), *IL6R*×LES (*β* = −0.32, *t* = 1.06, *p* = 0.30), *IL6*×*IL6R* (*β* = −0.13, *t* = −0.49, *p* = 0.62), and *IL6*×*IL6R*×LES (*β* = 0.37, *t* = −1.16, *p* = 0.25).

**Table S1. Correlations between IL-6 indices and quantitative variables (*n* = 108)**

| Variable | IL-6 diurnal index |  | IL-6 total output |  | Age |  | Years of education |  | BMI | Daily caffeine intake | Monthly alcohol  consumption | Sleep duration | Sleep quality |  | Perceived stress | Physical condition |
| --- | --- | --- | --- | --- | --- | --- | --- | --- | --- | --- | --- | --- | --- | --- | --- | --- |
| IL-6 diurnal index | − |  |  |  |  |  |  |  |  |  |  |  |  |  |  |  |
| IL-6 total output | 0.18 |  | − |  |  |  |  |  |  |  |  |  |  |  |  |  |
| Age | 0.32 | ^a^ | 0.20 | ^c^ | − |  |  |  |  |  |  |  |  |  |  |  |
| Years of education | -0.08 |  | 0.04 |  | 0.16 |  | − |  |  |  |  |  |  |  |  |  |
| BMI | 0.12 |  | 0.26 | ^b^ | 0.17 |  | -0.05 |  | − |  |  |  |  |  |  |  |
| Daily caffeine intake (mg) | 0.01 |  | 0.14 |  | 0.21 | ^c^ | 0.05 |  | -0.12 | − |  |  |  |  |  |  |
| Monthly alcohol consumption (unit) | -0.02 |  | -0.02 |  | 0.30 | ^b^ | 0.19 | ^c^ | -0.13 | 0.08 | − |  |  |  |  |  |
| Sleep duration | 0.11 |  | -0.20 | ^c^ | 0.06 |  | 0.06 |  | -0.05 | -0.07 | 0.03 | − |  |  |  |  |
| Sleep quality | 0.10 |  | -0.09 |  | -0.18 |  | 0.03 |  | -0.05 | 0.02 | -0.13 | 0.11 | − |  |  |  |
| Perceived stress | -0.04 |  | -0.13 |  | 0.07 |  | 0.03 |  | 0.01 | -0.03 | -0.01 | -0.04 | -0.22 | ^c^ | − |  |
| Physical condition | -0.18 |  | 0.00 |  | -0.10 |  | -0.01 |  | -0.04 | 0.07 | -0.01 | 0.03 | 0.24 | ^c^ | -0.02 | − |

^a^*p* < 0.001, ^b^*p* < 0.01, ^c^*p* < 0.05.

IL-6, interleukin-6; BMI, body mass index.

**Table S2. Menstruation status and IL-6 indices in female participants (*n* = 60)**

| Menstrual status | *n* | IL-6 diurnal index | | Total IL-6 output | |
| --- | --- | --- | --- | --- | --- |
|  |  | Mean | SD | Mean | SD |
| Follicular phase | 14 | 8.1 | 5.6 | 161.6 | 94.2 |
| Ovulatory phase | 10 | 6.5 | 10.6 | 217.6 | 158.0 |
| Luteal phase | 20 | 12.1 | 23.3 | 181.9 | 150.7 |
| During menstruation | 11 | 17.2 | 35.5 | 263.7 | 292.6 |
| Irregular cycle | 2 | 4.2 | 0.1 | 177.9 | 69.2 |
| Menopause | 3 | 15.8 | 2.4 | 190.1 | 3.5 |

No significant differences were found for both IL-6 indices: *F* (5, 54) = 0.85, *p* = 0.52 for IL-6 diurnal index; *F* (5, 54) = 0.48, *p* = 0.79 for total IL-6 output.

IL-6, interleukin-6.

**Table S3. Correlations between potential IL-6 diurnal indices and amygdala subnuclei activity to fearful (vs. neutral) faces**

| Variable | T5−T4 |  | T5−T3 |  | T1−T4 |  | L BLA |  | R BLA |  | L CEM |  | R CEM |
| --- | --- | --- | --- | --- | --- | --- | --- | --- | --- | --- | --- | --- | --- |
| T5−T4 | ― |  |  |  |  |  |  |  |  |  |  |  |  |
| T5−T3 | 0.76 | ^a^ | ― |  |  |  |  |  |  |  |  |  |  |
| T1−T4 | 0.18 | ^d^ | -0.05 |  | ― |  |  |  |  |  |  |  |  |
| L BLA | 0.31 | ^b^ | 0.27 | ^b^ | -0.16 |  | ― |  |  |  |  |  |  |
| R BLA | 0.10 |  | 0.10 |  | -0.09 |  | 0.55 | ^a^ | ― |  |  |  |  |
| L CEM | 0.25 | ^c^ | 0.20 | ^c^ | -0.22 | ^c^ | 0.65 | ^a^ | 0.45 | ^a^ | ― |  |  |
| R CEM | 0.02 |  | 0.05 |  | -0.08 |  | 0.46 | ^a^ | 0.56 | ^a^ | 0.57 | ^a^ | ― |

^a^*p* < 0.001, ^b^*p* < 0.01, ^c^*p* < 0.05, ^d^*p* < 0.1. Effects of age, sex, BMI, sleep duration, handedness, and the time interval between MRI and saliva collection were adjusted for in the analysis. Values of BLA and CEM are those extracted from the masks defined by Jüelich histological atlas (i.e., pre-defined, whole structural mask).

IL-6, interleukin-6; L, left; R, right; BLA, basolateral amygdala; CEM, centromedial amygdala; T1, time 1 (7:00); T3, time 3 (12:00); T4, time 4 (18:00), T5, time 5 (0:00).

**References**

1. Tietze S, Lincoln R. Fertility Regulation and the Public Health. New York, NY: Springer-Verlag; 1987.

2. Ogino K. Conception Period of Women. New York, NY: Medical Arts Publishing Company; 1934.

3. Baranger DAA, Margolis S, Hariri AR, Bogdan R. An earlier time of scan is associated with greater threat-related amygdala reactivity. Soc Cogn Affect Neurosci. 2017 Aug 01;12(8):1272-1283. eng. doi:10.1093/scan/nsx057. Cited in: Pubmed; PMID 28379578.
